# Retraction Note: Selective inheritance of target genes from only one parent of sexually reproduced F1 progeny in *Arabidopsis*

**DOI:** 10.1038/s41467-022-31001-3

**Published:** 2022-06-07

**Authors:** Tao Zhang, Michael Mudgett, Ratnala Rambabu, Bradley Abramson, Xinhua Dai, Todd P. Michael, Yunde Zhao

**Affiliations:** 1 Division of Biological Sciences, Section of Cell and Developmental Biology, University of California, San Diego, La Jolla, CA USA; 2 Plant Molecular and Cellular Biology Laboratory, The Salk Institute for Biological Studies, La Jolla, CA USA

**Keywords:** Genetic engineering, Molecular engineering in plants, Transgenic plants

Retraction to: *Nature Communications* 10.1038/s41467-021-24195-5, published online 22 June 2021.

We retract the article cited above because genotyping results from recent experiments are not consistent with the conclusions presented in the paper.

We recently genotyped a selection of F2 plants in order to identify plants to use for an introgression experiment.

In the original study, we confirmed homozygosity by using a pair of oligonucleotides covering the large gene drive region in its entirety, a region that is too large to be amplified by PCR if both alleles have integrated the gene drive element. When we analyzed F2 plants using a pair of oligonucleotides targeting a smaller region of the gene drive, we found a small fragment was amplified only in about 75% of the plants. The absence of the band in the remaining F2 plants can be accounted for by a large NHEJ based deletion in one of the alleles of the F1 plants, which can result in the removal of the oligonucleotide binding sites. Thus, the F1 plants presented in the paper are not homozygous as stated in the published paper.

In light of the new genotyping data that invalidate our conclusions on gene drives, we are retracting the paper. The gene targeting results of both the CRY1 lines and NPY5-GFP lines remain valid. We apologize for any inconvenience the publication of this work may have caused to the scientific community. All authors agree to the retraction.

